# Diagnostic performance of the time to positivity of blood cultures to distinguish true bacteremia from contaminants based on an automated system

**DOI:** 10.17843/rpmesp.2023.404.12724

**Published:** 2023-12-18

**Authors:** Aracely Laque-Ale, Miguel Hueda-Zavaleta, Juan Carlos Gómez de la Torre, Luis Alvarado, José Alonso Cáceres del Águila

**Affiliations:** 1 Faculty of Health Sciences, Tacna Private University, Tacna, Peru. Tacna Private University Faculty of Health Sciences UTacna Private University Tacna Peru; 2 Hospital III Daniel Alcides Carrión EsSalud, Tacna, Peru. Hospital III Daniel Alcides Carrión EsSalud Tacna Peru; 3 Roe Clinical Laboratory, Lima, Peru. Roe Clinical Laboratory Lima Peru

**Keywords:** Microbiological techniques, bacteremia, bacterial infections, mycoses, blood culture, diagnosis

## Abstract

**Objective.:**

To determine the diagnostic performance of blood culture positivity times for distinguishing true bacteremia from contaminants in the automated “BACT/ALERT®” system.

**Materials and methods.:**

A cross-sectional, diagnostic test-type study was conducted from a database of blood culture samples processed between January 2016 and August 2021. All blood culture samples from patients with suspected bacteremia were included; blood culture samples were entered into the “BACT/ALERT®” system to differentiate true bacteremia from contaminants.

**Results.:**

We obtained 33,951 blood cultures samples, of which 3875 were positive. Of the total number of positive blood cultures, 75.2% (n=2913) were true bacteremia and 24.8% (n=962) were contaminants. The median time to positivity in blood cultures with true bacteremia was significantly shorter (16.3 hours; IQR: 11.2 - 24.9) than the median time to positivity of blood cultures with contaminants (22.5 hours; IQR: 18.4 - 31.8; p<0.001). The positivity time showed the capacity to differentiate true bacteremia from contaminants, with an AUC-ROC of 0.73 (95%CI: 0.71 - 0.75), with 85% and 63% sensitivity and specificity respectively for the diagnosis of contaminants when the positivity time exceeds 16.5 hours. The use of antibiotics prior to sampling delayed the time to positivity, while having fever before sampling shortened the time to positivity.

**Conclusions.:**

Our results show good diagnostic performance of blood culture positivity times to differentiate true bacteremia from contaminants using the “BACT/ALERT®” system when the positivity time was longer than 16.5 hours.

## INTRODUCTION

Early identification of bacteremia is an important challenge for medical personnel, since it is considered a clinical and public health problem worldwide, not only as a pathological entity but also because of all the infectious and non-infectious complications it causes, being responsible for high morbidity and mortality rates ^1^^,^^2^. The current world mortality rate due to bacteremia ranges from 21 to 32 deaths per 100,000 inhabitants ^3^, which reflects the importance of its detection, based on the identification of viable bacteria in the patient’s bloodstream in blood cultures ^4^.

However, blood culture, considered the gold standard for the diagnosis of bacteremia ^5^, has limited usefulness due to its low sensitivity when diagnosing true bacteremia ^6^. This is due to the fact that not all detected bacteria are indicative of a real infection; in many cases, blood cultures may be contaminated with bacteria that do not cause true bacteremia. A poor technique of collection and preservation of the sample, in addition to the previous use of antibiotics during the collection, could influence the growth of microorganisms, causing contamination and alteration of the results of the blood culture ^7^^,^^8^.

Therefore, the diagnostic performance of blood cultures has improved significantly in the last decade and the development of rapid diagnostic tests and automated systems based on innovative technologies have progressed as well ^9^, which are useful for the early diagnosis of true bacteremia, through the detection of the time to positivity ^10^.

In this sense, the positivity time of blood cultures has been suggested as a useful indicator to differentiate true bacteremia from contaminating bacteremia ^11^. A rapid and accurate identification of the causative microorganism is very useful during clinical management, optimizing treatment, in addition to reducing unnecessary costs and resources in health systems ^12^. For this reason, this study aimed to determine the diagnostic performance of blood culture positivity times to distinguish true bacteremia from contaminants using the “BACT/ALERT®” automated system.

**KEY MESSAGES**

Motivation for the study: Determining the diagnostic performance of the time to positivity of blood cultures to distinguish true bacteremia from contaminants could be very useful to achieve an accurate and early diagnosis.

Main findings: Blood culture positivity times showed discriminant capacity to differentiate true bacteremia from contaminants, with an AUC-ROC of 0.73 (95%CI: 0.71 - 0.75), (S: 85%, E: 63%) for the diagnosis of contaminant blood cultures when the positivity time exceeds 16.5 hours. They also showed discriminant capacity for the diagnosis of coagulase-negative staphylococcus and *Candida* spp..

Implications: Defining the diagnostic utility of blood culture positivity times will help health personnel to make better decisions regarding patient treatment and thus avoid unnecessary hospital costs.

## MATERIALS AND METHODS

### Data source and sample

We carried out a diagnostic cross-sectional study from January to February 2023 in the city of Tacna, Peru, using a database of blood culture samples processed during the period from January 2016 to August 2021 in a private clinical laboratory in Lima, Peru.

The study included all blood culture samples from patients with suspected bacteremia that had issued a microorganism recognition alert by the automated system and that belonged to a set of blood cultures (minimum two bottles per patient). Blood culture samples that did not have complete data or did not meet the inclusion criteria were discarded.

Samples for blood cultures were collected by qualified personnel who followed biosafety protocols and applied the following recommendations: first the venipuncture site was disinfected with 70% alcohol or 2% chlorhexidine gluconate, then at least one set of two or more blood culture bottles with the required volume level was extracted. Subsequently, the blood culture samples were placed into the automated smart incubator “BACT/ALERT®” (BioMériux, Durham, NC, USA). In order to measure the time of blood culture positivity, we recorded the time each time an alarm was detected, which indicated growth in a blood culture bottle. An aliquot was then collected from these bottles for Gram staining and subculturing on chocolate, blood, MacConkey and Sabouraud agar. Finally, the Vitek 2.0 automated system and MALDI-TOF MS (bioMérieux, Marcy l'Etoile, France) were used for identification and antimicrobial susceptibility.

BACT/ALERT® is an incubator with an intelligent automated continuous microbial detection system, which allows the identification of a wide variety of bacterial and fungal microorganisms. This system detects the increase of CO_2_ produced during microbial growth, which causes a colorimetric change in its base sensor, increasing the amount of reflected light. This increase in brightness triggers a visual and audible alarm for positive bottles in an automated manner.

We used the reported sensitivity and specificity of blood culture positivity time to predict contaminating blood cultures from the study by Ruiz-Giardín *et al.*^13^ in order to calculate the diagnostic accuracy and sample size. That study reported a sensitivity of the time to positivity (>14.7 hours) of 90% and a specificity of 63%. With these parameters, a confidence level of 95%, 3875 blood cultures included in our sample size, and a contaminant prevalence of 25%, the diagnostic accuracy was calculated to be 1.89% for sensitivity and 1.75% for specificity.

### Variables

The variable time to positivity was defined as the hours elapsed from sample collection to the appearance of the alarm signal in the intelligent incubator. On the other hand, a blood culture with true bacteremia was defined when at least one bottle of the blood culture set isolated a gram-negative, fungal or gram-positive microorganism (2 or more bottles of positive blood cultures with the same microorganism were required if a coagulase-negative staphylococcus was identified). In contrast, we identified a blood culture with contaminant when one of the following microorganisms was isolated in only one bottle of the blood culture set: coagulase-negative staphylococcus, *Cutibacterium acnes* or *Corynebacterium*. The variable, “fever at the time of blood culture”, was considered positive when a temperature higher than 38.3°C was reported at the time of taking the blood culture sample. The variable, “received antibiotics prior to blood culture”, was considered positive when consumption of any antibiotic in the 48 hours prior to blood culture sampling was reported.

### Procedures

We used data recorded in the microbiology laboratory system from blood culture samples processed from January 2016 to August 2021. Once identified, those positive blood culture results were selected through a Microsoft Excel spreadsheet (version 16), with the aim of collecting information on the times of positivity and isolated microorganisms. Two researchers were responsible for the collection, analysis and creation of a database. One researcher contributed with the first review of the data analysis. Two researchers were in charge of a final quality control, in addition to the final revision and writing.

### Statistical analysis

Statistical analysis was performed with STATA v17.0 software (StataCorp., College Station, TX, USA). Categorical variables were presented as absolute and relative frequencies, and were compared using the Chi^2^ or Fisher’s exact test. Quantitative variables were presented as median and interquartile range (IQR), due to their non-normal distribution, and were compared by Mann Whitney U test. Finally, we evaluated the prognostic ability of blood culture positivity times, as predictors of true bacteremia vs. contaminants, using ROC (Receiver Operating Characteristic) curves, ROC area under the curve (AUC); cut-off points were selected according to the highest Youden index.

### Ethical Aspects

This research follows the Helsinki norms for research in human beings. The protocol was approved by the Comité Institucional de Ética en Investigación Tacna (CIEI) of the Red Asistencial Essalud Tacna (CA N°003-2023). Informed consent was not requested due to the retrospective and observational nature of the study.

## RESULTS

**Figure 1:**
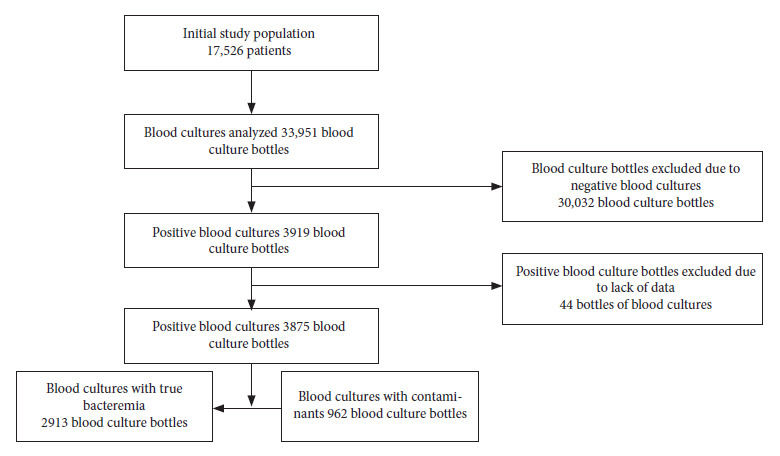
Sampling flow diagram**.**

We obtained 33,951 blood culture bottles from 17,526 patients. Of these, 30,032 blood culture bottles were negative, and 3919 were positive, of which 44 were excluded due to lack of data. A total of 3875 positive blood culture bottles from 1251 patients were included (Figure 1). The median age of the patients was 59 years (IQR: 41-73). Of the total positive blood cultures, 75.1% (n= 2913) were blood cultures with true bacteremia and 24.8% (n= 962) were contaminating blood cultures (Table 1).

**Table 1:** Characteristics of the study population and times of blood culture positivity with true bacteremia and with contaminants.

Variable	Total (N=3875) n(%)	Contaminants (N=962) n(%)	True bacteriemia (N=2913) n(%)	p-value
Age (years) ^a^	59 (41 - 73)	57 (30 - 72)	59 (42 - 74)	<0,001 ^b^
Positivity time in hours ^a^	18.4 (12.0 - 27.3)	22.5 (18.4 - 31.8)	16.3 (11.2 - 24.9)	<0,001 ^b^
Positivity in <24 hours				<0,000 ^c^
Yes	1671 (43.1)	296 (30.8)	1375 (47.2)	
No	2204 (56.9)	666 (69.2)	1538 (52.8)	
Fever at time of blood culture (n= 441)				0,254 ^d^
Yes	376 (85.3)	11 (73.3)	365 (85.7)	
No	65 (14.7)	4 (26.7)	61 (14.3)	
Time to positivity in hours in patients with fever ^a^	13.9 (10.6 - 18.9)	18.8 (15.8 - 25.7)	13.3 (10.5 - 18.5)	<0,001 ^b^
Received antibiotics prior to blood cultures (n=440)				0,713 ^d^
Yes	410 (93.2)	31 (96.9)	379 (92.9)	
No	30 (6.8)	1 (3.1)	29 (7.1)	
Time to positivity in hours in patients who received antibiotics ^a^	14.2 (11.1 - 20.1)	20.0 (15.8 - 36.5)	13.8 (10.8 - 18.9)	<0,001 ^b^

a Median and interquartile range; ^b^ Mann Whitney U; ^c^ Chi^2^; ^d^ Fisher’s exact test.

### Microorganisms

Of the 2913 positive bottles for true bacteremia, the most frequently isolated microorganisms were: coagulase-negative staphylococcus (22.9%), *Escherichia coli* (14.0%), *Klebsiella* spp. (10.2%), *Enterococcus* (6.6%), *Staphylococcus aureus* (6.3%), Candida (6.1%), *Pseudomonas* (5.3%), *Streptococcus* (4.9%), *Enterobacter* spp. (4.8%), *Acinetobacter* spp. (4.6%) and *Stenotrophomonas maltophilia* (2.1%) (Table 2).

**Table 2:** Frequency, percentage and time of positivity of main microorganisms detected in blood cultures with true bacteremia (N=2913).

Microorganisms	True bacteriemia n(%)	Positivity time in hours Median (IQR)
Gram positive		
Coagulase-negative Staphylococcus	666 (22.9)	21.6 (17.9 - 29.6)
*Enterococcus* spp.	191 (6.6)	12.8 (10.9 - 16.0)
*Staphylococcus aureus*	183 (6.3)	16.7 (11.4 - 23.9)
*Streptococcus* spp.	142 (4.9)	12.7 (8.5 - 12.5)
*Streptococcus pneumoniae*	24 (0.8)	7.9 (6.3 - 10.5)
*Candida* spp.	177 (6.1)	38.6 (28.7 - 57.9)
Gram negative		
*Echerichia coli*	408 (14.0)	10.7 (8.7 - 12.6)
*Klebsiella* spp.	298 (10.2)	11.6 (8.7 - 13.8)
*Pseudomonas* spp.	154 (5.3)	16.5 (13.9 - 22.3)
*Enterobacter* spp.	140 (4.8)	11.7 (8.9 - 14.3)
*Acinetobacter* spp.	133 (4.6)	12.6 (9.7 - 16.4)
*Salmonella* spp.	88 (3.0)	14.5 (11.2 - 19.6)
*Stenotrophomonas maltophilia*	62 (2.1)	23.9 (15.4 - 31.4)
*Serratia*	38 (1.3)	18.9 (12.7 - 32.7)
*Proteus*	12 (0.4)	18.3 (17.5 - 29.2)
Other	197 (6.8)	NA

NA: not applicable (because they are grouped microorganisms).

Of the 962 contaminated blood culture bottles, the most frequently isolated microorganisms were coagulase-negative Staphylococcus (98.7%), *Corynebacterium* (0.6%), and *Cutibacterium acnes* (0.6%).

### Time to positivity

**Figure 2:**
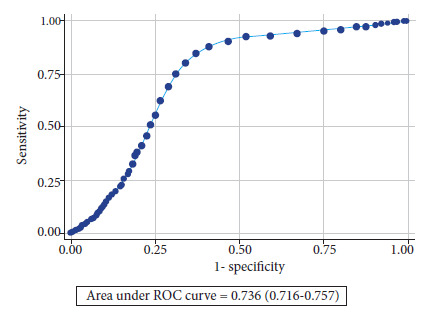
ROC curve and AUC ROC of the time to positivity of cultures to differentiate true bacteremia from contaminants.

The median time to positivity in blood cultures with true bacteremia was statistically lower than in blood cultures with contaminants (16.3; IQR: 11.2 - 24.9 vs. 22.5; IQR: 18.4 - 31.8 hours; p<0.001). We found that 73.4%, 87.1%, 92.2%, 97.1%, 99.2% and 100% of blood cultures with true bacteremia grew in the first 24, 36, 48, 48, 72, 96 and 120 hours, respectively. The time to positivity of blood cultures showed discriminant capacity to differentiate true bacteremia from contaminating blood cultures, with an AUC-ROC of 0.73 (95%CI: 0.71 - 0.75), a sensitivity of 85% and specificity of 63% for the diagnosis of contaminating blood cultures when the time to positivity was greater than 16.5 hours (Figure 2). We also found discriminating ability of blood culture positivity time for the diagnosis of coagulase-negative staphylococcal bacteremia (AUC-ROC: 0.72; sensitivity: 84.75%; specificity: 62.05%; cut-off point ≥16.5 hours), *stenotrophomonas maltophilia* (AUC-ROC: 0.61; sensitivity: 50.0%; specificity: 69.97%; cut-off point ≥24.5 hours) and *Candida* spp. fungemia (AUC-ROC: 0.61; sensitivity: 50.0%; specificity: 69.97%; cut-off point ≥24.5 hours) (Figure 3). (AUC-ROC: 0.79; sensitivity: 72.9%; specificity: 83.3%; cutoff point ≥31.5 hours) (Table 3).

**Table 3:** Area under the ROC curve and cut-off points with the highest AUC ROC of positivity times to differentiate blood cultures with contaminants from true bacteremia (N=3875).

Variable	AUC-ROC (95%CI)	Sensibility (%)	Specificity (%)	LHR (+)	LHR (-)	Youden index
Time to positivity in blood culture hours for predicting contaminants	0.74 (0.72 - 0.76)	85.0	63.0	2.30	0.23	16.5
Specific microorganisms						
Coagulase-negative Staphylococcus	0.72 (0.70 - 0.74)	84.8	62.1	2.23	0.24	16.5
*Stenotrophomonas maltophilia*	0.61 (0.53 - 0.70)	50.0	70.0	1.66	0.71	24.5
*Candida* spp.	0.80 (0.75 - 0.84)	72.9	83.3	4.36	0.32	31.5

AUC: area under the curve; ROC: receiver operating characteristic; LHR: Likelihood ratio; 95%CI: 95% confidence interval.

### Fever and antibiotics at the time of blood culture sample collection

We were able to obtain information from a subpopulation of the study (n=440), of which 410/440 patients received antibiotics prior to blood culture collection; these patients had longer positive times than those who did not receive antibiotics previously (14.2 hours, IQR: 11.1 - 20.1 and 10.8 hours, IQR: 9.4 - 14.2; p=0.004, respectively). Likewise, in a subpopulation of the study (n=441), 376/441 patients had fever (temperature >38.3°C) at the time of blood culture collection, noting that the median positivity time of the blood cultures was lower than in those who did not had fever at the time of sample collection (13.9 hours, IQR: 10.6 - 18.9 and 14.9 hours, IQR: 11.8 - 31.6; p=0.039, respectively).

## DISCUSSION

We found that the median time to positivity in blood cultures with true bacteremia was statistically significantly lower than in blood cultures with contaminants. Furthermore, our findings show that a blood culture positivity time greater than 16.5 hours can predict the presence of blood cultures with contaminants, with a sensitivity and specificity of 85% and 63%, respectively. Likewise, the positivity time showed discriminant capacity to identify candidemia (sensitivity: 73%; specificity: 83%) and bacteremia due to *Stenotrophomonas maltophilia* (sensitivity: 50%; specificity: 70%).

Time to blood culture positivity can help distinguish between true bacteremia and contaminating microorganisms. Ruiz-Giardín JM, *et al*. reported that a blood culture positivity time greater than 14.7 hours had a sensitivity of 90% and a specificity of 63% with an AUC/ROC of 0.80 for predicting the detection of blood cultures with contaminants ^13^. Similarly, Morioka S. *et al*. reported that positivity time greater than 20 hours had a sensitivity of 86% for predicting coagulase-negative staphylococcal contamination ^14^. The time of positivity of blood cultures is thought to be an indicator of bacterial load. Some studies have described an inverse correlation between time to positivity and bacterial concentration in blood cultures ^15^. Previous reports show that antibiotic treatment prior to the blood culture delays the time to positivity ^13^^,^^16^^-^^18^, probably secondary to the effect of reducing the bacterial concentration. Likewise, a very short positivity time in *K. pneumoniae* bacteremia was associated with higher mortality ^19^, we presume that this association with mortality is due to a high bacterial load.

Most studies found that between 93 and 98% of true bacteremia cases were detected in the first 48 hours of incubation ^17^^,^^20^^-^^23^, and only 0.1% of true bacteremia cases were isolated after 4 days of incubation ^24^. In our study, 92.2%, 99.2%, and 100% of true bacteremia cases were isolated within less than 2, 4, and 5 days to positivity, respectively. These findings suggest that, with the BACT/ALERT® kit, an incubation time of 4 days could be sufficient for the diagnosis of true bacteremia, which would reduce the number of isolations of contaminating microorganisms, which have been associated with increased hospital costs ^25^^,^^26^. Similar findings have been reported with other automated incubator systems such as BACT/ALERT® virtuo ^24^, Difco ESP ^27^^)^ and Accumed ESP-384 ^28^.

The time elapsed until blood culture positivity has been suggested as an antimicrobial control strategy. Pardo *et al*. detected *Pseudomonas aeruginosa*, *E. Coli*, *K. pneumoniae* and *E. cloacae* in the first 48 hours of incubation ^17^, and proposed that, if a blood culture is negative at 48 hours, the probability of it being a true bacteremia is minimal, due to its high negative predictive value, very similar to the negative blood culture at 5 days; this could be an opportunity to reduce the unnecessary use of antibiotics mainly when the patient is stable and has not received antibiotics prior to the blood culture. Similar findings were reported by other authors ^13^^,^^24^.

We found differences in blood culture positivity times according to the isolated microorganism, mainly in two relevant pathogens: *Candida* and *Stenotrophomonas maltophilia*, which had positivity times of 38.61 and 23.90 hours respectively, such that, if blood cultures were positive beyond 24 hours of incubation, the possibility of isolating *Stenotrophomonas maltophilia* was 50%; on the other hand, if blood cultures were positive beyond 39 hours, the possibility of isolating *Candida* was 50%. This could be of particular relevance when deciding on therapy, since due to the multiple mechanisms of intrinsic resistance to antibiotics that *Stenotrophomonas* possesses, there is a high possibility of initiating inappropriate antibiotic therapy, which is associated with higher mortality (61% vs. 30%) and worse outcomes ^29^^,^^30^. Likewise, it is possible that the etiology of bacteremia may be different according to age groups, and this may also influence the time of blood culture positivity. In contrast to our study, which included patients ≥18 years of age, the median blood culture positivity times were 11.2 hours and 12.6 hours in pediatric patients aged 0 to 1 year and 1 to 15 years, respectively ^21^. This positivity time is lower than that found in our study, in which most patients were adults.

Some inflammatory markers have been evaluated to predict true bacteremia in positive blood cultures. Procalcitonin had the best performance with an AUC ROC of 0.79, sensitivity of 76% and specificity of 69% with a cut-off point of 0.5 ng/ml ^31^. Followed by C-reactive protein, with an AUC ROC of CRP of 0.64 and sensitivity of 87% at a cutoff point of 9 mg/l; and leukocytosis >12,000/mm^3^ with an AUC ROC of 0.69, and a sensitivity of 65.5% ^32^. Therefore, compared to these inflammatory markers, the time to blood culture positivity found in our study (AUC ROC of 0.74, sensitivity of 85% and specificity of 63%) could be a better predictor of true bacteremia than C-reactive protein and leukocytosis, and similar to procalcitonin.

This study has certain limitations. The main limitation was the retrospective nature of the study, since it did not allow us to determine other variables such as severity of the condition, focus of infection, antibiotics and outcome, among others. Nor was it possible to quantify by weight the volume of blood inoculated in the blood culture bottles and how much was inoculated, the time elapsed between blood collection and inoculation, in addition to the type of antibiotic, dose and treatment time, which may affect its performance. Another limitation was that approximately 3434 samples did not have information regarding the variable “fever at the time of blood culture” and it is possible that a large percentage of these may or may not have presented fever, which could affect the results. Likewise, there is no information for the majority of the population regarding the use of antibiotics before blood culture, which could greatly affect the results. Finally, all blood cultures were analyzed with the BacT Alert kit and these findings should not be extrapolated to other automated kits.

This study demonstrated that blood culture positivity times can predict true contaminant bacteremia when the positivity time is greater than 16.5 hours. Likewise, the positivity time proved to be useful for predicting bacteremia due to *Candida* and *Stenotrophomonas maltophilia*. Understanding the diagnostic performance of this method would help health personnel to make better decisions regarding patient treatment and avoid unnecessary hospital costs.
