# Supplementary material for: Diagnostic performance of the time to positivity of blood cultures to distinguish true bacteremia from contaminants based on an automated system
Source: Rev Peru Med Exp Salud Publica. 2023 Dec 18;40(4):451–8. doi: 10.17843/rpmesp.2023.404.12724 (PMC11138833; doi:10.17843/rpmesp.2023.404.12724)
Supplement: Translation

# Translation: Utilidad diagnóstica de los tiempos de positividad de hemocultivos para distinguir verdaderas bacteriemias de contaminantes en base a un sistema automatizado

**Authors:** Aracely Laque-Ale, Miguel Hueda-Zavaleta, Juan Carlos Gómez de la Torre, Luis Alvarado, José Alonso Cáceres del Águila

**Affiliations:** 1 Facultad de Ciencias de la Salud, Universidad Privada de Tacna, Tacna, Perú.; 2 Hospital III Daniel Alcides Carrión EsSalud, Tacna, Perú.; 3 Laboratorio Clínico Roe, Lima, Perú.

**Keywords:** Técnicas microbiológicas, bacteriemia, infecciones bacterianas, micosis, cultivo de sangre, diagnóstico

## Abstract

**Objetivo.:**

Determinar la utilidad diagnóstica de los tiempos de positividad de hemocultivos para distinguir verdaderas bacteriemias de contaminantes en el sistema automatizado «BACT/ALERT®».

**Materiales y métodos.:**

Se realizó un estudio transversal de tipo pruebas diagnósticas, a partir de una base de datos de muestras de hemocultivos procesadas entre enero del 2016 y agosto del 2021. Se incluyeron todas las muestras de hemocultivos de pacientes con sospecha de bacteriemia, las muestras de hemocultivos fueron ingresadas al sistema «BACT/ALERT®» para diferenciar verdaderas bacteriemias de contaminantes.

**Resultados.:**

Se analizó un total de 33 951 frascos de hemocultivos y se obtuvieron 3875 frascos positivos. El 75,2% (n=2913) del total de hemocultivos positivos fueron verdaderas bacteriemias y 24,8% (n=962) fueron contaminantes. La mediana de tiempo de positividad en los hemocultivos con verdaderas bacteriemias fue significativamente menor (16,3 horas; RIC: 11,2 - 24,9) que la mediana de tiempo de positividad de hemocultivos con contaminantes (22,5 horas; RIC: 18,4 - 31,8; p<0,001). El tiempo de positividad demostró capacidad discriminante para diferenciar verdaderas bacteriemias de contaminantes, con un AUC-ROC de 0,73 (IC95%: 0,71 - 0,75), con 85% y 63% de sensibilidad y especificidad respectivamente para el diagnóstico de contaminantes cuando el tiempo de positividad supera las 16,5 horas. La administración de antibióticos previos a la toma retrasó el tiempo de positividad, en cambio, haber presentado fiebre antes de la toma de muestra acortó el tiempo de positividad.

**Conclusiones.:**

Nuestros resultados muestran un buen desempeño de los tiempos de positividad de hemocultivos para diferenciar verdaderas bacteriemias de contaminantes utilizando el sistema «BACT/ALERT®» cuando el tiempo de positividad fue superior a 16,5 horas.

## INTRODUCCIÓN

La identificación temprana de las bacteriemias es un reto importante para el personal médico, ya que es considerada un problema clínico y de salud pública a nivel mundial, no solo como entidad patológica propia sino por las diversas complicaciones infecciosas y no infecciosas que esta produce, siendo responsable de tasas elevadas de morbimortalidad ^1^^,^^2^. La tasa actual de mortalidad mundial por bacteriemia varia de 21 a 32 muertes por 100 000 habitantes ^3^, dato que refleja la importancia de su detección, basándose en la identificación de bacterias viables en el torrente sanguíneo del paciente, lo que se logra mediante la realización de hemocultivos ^4^.

Sin embargo, el hemocultivo, considerado como el gold estándar para el diagnóstico de bacteriemias ^5^, presenta utilidad limitada por su baja sensibilidad al momento de diagnosticar verdaderas bacteriemias ^6^. Esto debido a que no todas las bacterias detectadas indican una infección real; en muchos casos, los hemocultivos pueden estar contaminados con bacterias que no causan una verdadera bacteriemia. Una mala técnica de recolección y conservación de la muestra, además del uso previo de antibióticos durante la toma, podrían influenciar en el crecimiento de los microorganismos, generando contaminación y alteración de los resultados del hemocultivo ^7^^,^^8^.

Por ende, en la última década se han realizado mejoras importantes en cuanto al rendimiento diagnóstico de los hemocultivos, además de progresos en el desarrollo de pruebas diagnósticas rápidas y sistemas automatizados que se basan en tecnologías innovadoras ^9^, siendo útiles para diagnosticar precozmente una verdadera bacteriemia, a través de la detección del tiempo de positividad ^10^.

En ese sentido, el tiempo de positividad de los hemocultivos se ha sugerido como un indicador útil para diferenciar las verdaderas bacteriemias de las contaminantes ^11^. Una identificación rápida y precisa del microorganismo causante resulta ser de mucha utilidad durante el manejo clínico, optimizando el tratamiento, además de reducir costos y recursos innecesarios en los sistemas de salud ^12^. Por tal motivo, el objetivo de este estudio fue determinar la utilidad diagnóstica de los tiempos de positividad de hemocultivos como para distinguir verdaderas bacteriemias de contaminantes utilizando el sistema automatizado «BACT/ALERT®».

**MENSAJE CLAVE**

Motivación para realizar el estudio: Determinar la utilidad diagnóstica de los tiempos de positividad de hemocultivos para distinguir verdaderas bacteriemias de contaminantes podría resultar de gran utilidad para lograr un diagnóstico certero y precoz.

Principales hallazgos: Los tiempos de positividad de hemocultivos mostraron capacidad discriminante para diferenciar verdaderas bacteriemias de contaminantes, con un AUC-ROC de 0,73 (IC95%: 0,71 - 0,75), (S: 85%, E: 63%) para el diagnóstico de hemocultivos contaminantes cuando el tiempo de positividad supera las 16,5 horas. También demostraron capacidad discriminante para el diagnóstico de estafilococo coagulasa negativo y *Candida s*pp..

Implicancias: Conocer la utilidad diagnóstica de los tiempos de positividad de hemocultivos ayudará al personal de salud a tomar mejores decisiones respecto al tratamiento del paciente y así evitar costos hospitalarios innecesarios.

## MATERIALES Y MÉTODOS

### Fuente de datos y muestra

Se realizó un estudio transversal de tipo pruebas diagnósticas, que se ejecutó de enero a febrero del 2023 en la ciudad de Tacna, Perú, a partir de una base de datos de muestras de hemocultivos procesadas durante el periodo desde enero 2016 a agosto 2021 en un laboratorio clínico privado de Lima, Perú.

Se incluyeron en el estudio todas las muestras de hemocultivos de pacientes con sospecha de bacteriemia, que hayan emitido una alerta de reconocimiento de microorganismo por el sistema automatizado y que pertenecían a un set de hemocultivos (mínimo dos frascos por paciente). Se descartaron aquellas muestras de hemocultivos que no contaron con datos completos, o no cumplieron los criterios de inclusión.

Las muestras para los hemocultivos fueron recolectadas por personal calificado bajo protocolos de bioseguridad, aplicando las siguientes recomendaciones: primero se desinfectó la zona de venopunción con alcohol al 70% o gluconato de clorhexidina al 2%, luego se extrajo al menos un set de dos o más frascos de hemocultivos con el nivel de volumen requerido. Posteriormente, las muestras de hemocultivos fueron ingresadas al incubador inteligente automatizado «BACT/ALERT®» (BioMériux, Durham, NC, EE.UU.). Para medir el tiempo de positividad de los hemocultivos se registró el tiempo cada vez que una alarma fue detectada, la cual indicaba el crecimiento en un frasco de hemocultivo. Luego se procedió a recolectar una alícuota de estos frascos para aplicar la tinción de Gram y realizar subcultivos en agar chocolate, sangre, MacConkey y Sabouraud. Finalmente se utilizó el sistema automatizado Vitek 2,0 y MALDI-TOF MS (bioMérieux, Marcy l’Etoile, France) para la identificación y susceptibilidad antimicrobiana.

«BACT/ALERT®» es un incubador con sistema inteligente automatizado de detección microbiana continua, que permite identificar una gran variedad de microorganismos bacterianos y fúngicos. El sistema detecta el aumento de CO_2_ producido durante el crecimiento microbiano, lo que ocasiona un cambio colorimétrico en su sensor de base, incrementando la cantidad de luz reflejada. Este aumento de luminosidad genera una alarma visual y sonora para los frascos positivos de forma automatizada.

Para el cálculo de la precisión diagnóstica y tamaño muestral, se utilizó la sensibilidad y especificidad reportada del tiempo de positividad de hemocultivos para predecir hemocultivos contaminantes del estudio de Ruiz-Giardín *et al.*^13^. En su estudio, la sensibilidad del tiempo de positividad (>14,7 horas) fue de 90% y la especificidad fue de 63%. Con estos parámetros, un nivel de confianza del 95%, y 3875 hemocultivos incluidos en nuestro tamaño muestral, y una prevalencia de contaminantes de 25%, se calculó la precisión diagnóstica de 1,89% para la sensibilidad y de 1,75% para la especificidad.

### Variables

La variable tiempo de positividad fue definida como las horas transcurridas desde la toma de muestra hasta la aparición de la señal de alarma del incubador inteligente. Por otro lado, un hemocultivo con verdadera bacteriemia fue definido cuando al menos en un frasco del set de hemocultivos se aisló un microorganismo gram negativo, hongos o gram positivo (si se identificó un estafilococo coagulasa negativo, se requirió 2 o más frascos de hemocultivos positivos con el mismo microorganismo). En cambio, se identificó un hemocultivo con contaminante cuando se aisló en solo un frasco del set de hemocultivos alguno de los siguientes microorganismos: estafilococos coagulasa negativos, *Cutibacterium acnes* o *Corynebacterium*. La variable, “fiebre al momento del hemocultivo”, se consideró positiva cuando se reportó temperatura mayor a 38,3°C al momento de tomar la muestra para el hemocultivo. La variable, “recibió antibióticos previos a la toma de hemocultivos”, fue considerada positiva cuando se reportó el consumo de cualquier antibiótico en las 48 horas previas a la toma de la muestra para el hemocultivo.

### Procedimientos

Se utilizaron los datos registrados en el sistema del laboratorio de microbiología correspondientes a las muestras de hemocultivos procesadas desde enero 2016 a agosto del 2021. Una vez identificados, se seleccionaron a aquellos resultados de hemocultivos positivos a través de una hoja de cálculo de Microsoft Excel (versión 16), con el objetivo de recabar la información de los tiempos de positividad y microorganismos aislados. Dos investigadores se encargaron de la recolección, análisis y creación de una base de datos. Un investigador contribuyó con la primera supervisión del análisis de datos. Dos investigadores se encargaron de un último control de calidad, además de la revisión y redacción final.

### Análisis estadístico

El análisis estadístico fue realizado con el software STATA v17.0 (StataCorp., College Station, TX, EE. UU.). Las variables categóricas fueron presentadas como frecuencias absolutas y relativas, y se compararon usando el Test de Chi^2^ o exacta de Fisher. Las variables cuantitativas fueron presentadas como mediana y rango intercuartílico (RIC), debido a su distribución no normal, y se compararon mediante U de Mann Whitney. Finalmente, evaluamos la capacidad pronóstica de los tiempos de positividad de hemocultivos, como predictores de verdaderas bacteriemias vs contaminantes, mediante curvas ROC (Característica Operativa del Receptor), el área bajo la curva (AUC) ROC; los puntos de corte fueron seleccionados según el índice de Youden más alto.

### Aspectos éticos

Esta investigación sigue las normas de Helsinki para la investigación en seres humanos. El protocolo fue aprobado por el Comité Institucional de Ética en Investigación Tacna (CIEI) de la Red Asistencial Essalud Tacna (CA N°003-2023). No se solicitó el consentimiento informado debido a la naturaleza retrospectiva y observacional del estudio.

## RESULTADOS

Se obtuvieron 33 951 frascos de hemocultivos de 17 526 pacientes. De estos, 30 032 frascos de hemocultivos fueron negativos, y 3919 positivos, de los cuales 44 fueron excluidos por falta de data. Se incluyeron un total de 3875 frascos de hemocultivos positivos, provenientes de 1251 pacientes (Figura 1). La mediana de edad de los pacientes fue de 59 años (RIC: 41-73). Del total de hemocultivos positivos, el 75,1% (n= 2913) fueron hemocultivos con verdaderas bacteriemias y 24,8% (n= 962) fueron hemocultivos contaminantes (Tabla 1).

**Figura 1:** Diagrama de flujo de la toma de muestras.

**Tabla 1:** Características de la población en estudio y tiempos de positividad de hemocultivos con verdadera bacteriemia y con contaminantes.

Variable	Total (N=3875) n(%)	Contaminantes (N=962) n(%)	Verdadera Bacteriemia (N=2913) n(%)	Valor de p
Edad (años) ^a^	59 (41 - 73)	57 (30 - 72)	59 (42 - 74)	<0,001 ^b^
Tiempo de positividad en horas ^a^	18,4 (12,0 - 27,3)	22,5 (18,4 - 31,8)	16,3 (11,2 - 24,9)	<0,001 ^b^
Positividad en <24 horas				<0,000 ^c^
Sí	1671 (43,1)	296 (30,8)	1375 (47,2)	
No	2204 (56,9)	666 (69,2)	1538 (52,8)	
Fiebre al momento de toma de hemocultivo (n= 441)				0,254 ^d^
Sí	376 (85,3)	11 (73,3)	365 (85,7)	
No	65 (14,7)	4 (26,7)	61 (14,3)	
Tiempo de positividad en horas en pacientes con fiebre^a^	13,9 (10,6 - 18,9)	18,8 (15,8 - 25,7)	13,3 (10,5 - 18,5)	<0,001 ^b^
Recibió antibióticos previo a la toma de hemocultivo (n=440)				0,713 ^d^
Sí	410 (93,2)	31 (96,9)	379 (92,9)	
No	30 (6,8)	1 (3,1)	29 (7,1)	
Tiempo de positividad en horas en paciente que recibieron antibióticos ^a^	14,2 (11,1 - 20,1)	20,0 (15,8 - 36,5)	13,8 (10,8 - 18,9)	<0,001 ^b^

a Mediana y rango intercuartílico; ^b^ U de Mann Whitney; ^c^ Chi^2^; ^d^ Exacta de Fisher.

### Microorganismos

De los 2913 frascos positivos por verdaderas bacteriemias, los microorganismos más frecuentemente aislados fueron: estafilococo coagulasa negativo (22,9%), *Escherichia coli* (14,0%), *Klebsiella* spp. (10,2%), *Enterococcus* (6,6%), *Staphylococcus aureus* (6,3%), *Candida* (6,1%), *Pseudomonas* (5,3%), *Estreptococcus* (4,9%), *Enterobacter* spp. (4,8%), *Acinetobacter* spp. (4,6%) y *Stenotrophomonas maltophilia* (2,1%) (Tabla 2).

**Tabla 2:** Frecuencia, porcentaje y tiempo de positividad de principales microorganismos detectados en hemocultivos con verdadera bacteriemia (N=2913).

Microorganismos	Verdadera Bacteriemia n(%)	Tiempo de positividad en horas Mediana (RIC)
Gram positivos		
Estafilococo coagulasa negativo	666 (22,9)	21,6 (17,9 - 29,6)
*Enterococcus* spp.	191 (6,6)	12,8 (10,9 - 16,0)
*Estafilococo ureus*	183 (6,3)	16,7 (11,4 - 23,9)
*Streptococcus* spp.	142 (4,9)	12,7 (8,5 - 12,5)
*Streptococcus pneumoniae*	24 (0,8)	7,9 (6,3 - 10,5)
*Candida* spp.	177 (6,1)	38,6 (28,7 - 57,9)
Gram negativos		
*Echerichia coli*	408 (14,0)	10,7 (8,7 - 12,6)
*Klebsiella* spp.	298 (10,2)	11,6 (8,7 - 13,8)
*Pseudomonas* spp.	154 (5,3)	16,5 (13,9 - 22,3)
*Enterobacter* spp.	140 (4,8)	11,7 (8,9 - 14,3)
*Acinetobacter* spp.	133 (4,6)	12,6 (9,7 - 16,4)
*Salmonella* spp.	88 (3,0)	14,5 (11,2 - 19,6)
*Stenotrophomonas maltophilia*	62 (2,1)	23,9 (15,4 - 31,4)
*Serratia*	38 (1,3)	18,9 (12,7 - 32,7)
*Proteus*	12 (0,4)	18,3 (17,5 - 29,2)
Otros	197 (6,8)	NA

NA: no aplica (por ser microorganismos agrupados).

De los 962 frascos de hemocultivos contaminados, los microorganismos más frecuentemente aislados fueron estafilococo coagulasa negativo (98,7%), *Corynebacterium* (0,6%), y *Cutibacterium acnes* (0,6%).

### Tiempos de positividad

La mediana de tiempo de positividad en los hemocultivos con verdadera bacteriemia fue estadisticamente inferior que en los hemocultivos con contaminantes (16,3; RIC: 11,2 - 24,9 vs 22,5; RIC: 18,4 - 31,8 horas; p<0,001). El 73,4%, 87,1%, 92,2%, 97,1%, 99,2% y 100% de hemocultivos con verdaderas bacteriemias crecieron en las primeras 24, 36, 48, 72, 96 y 120 horas, respectivamente. El tiempo de positividad de los hemocultivos presentó capacidad discriminante para diferenciar verdaderas bacteriemias, de hemocultivos contaminantes, con un AUC-ROC de 0,73 (IC95%: 0,71 - 0,75), una sensibilidad de 85% y especificidad de 63% para el diagnóstico de hemocultivos contaminantes cuando el tiempo de positividad fue superior a las 16,5 horas (Figura 2). También se observó capacidad discriminante del tiempo de positividad en los hemocultivos para el diagnóstico de bacteriemia por estafilococo coagulasa negativo (AUC-ROC: 0,72; sensibilidad: 84,75%; especificidad: 62,05%; punto de corte ≥16,5 horas), *stenotrophomonas maltophilia* (AUC-ROC: 0,61; sensibilidad: 50,0%; especificidad: 69,97%; punto de corte ≥24,5 horas) y fungemia por *Candida* spp. (AUC-ROC: 0,79; sensibilidad: 72,9%; especificidad: 83,3%; punto de corte ≥31,5 horas) (Tabla 3).

**Figura 2:** Curva ROC y AUC ROC del tiempo de positividad de hemocultivos para diferenciar verdaderas bacteriemias de contaminantes.

**Tabla 3:** Área bajo la curva ROC y puntos de corte con mayor AUC ROC de los tiempos de positividad para diferenciar hemocultivos con contaminantes de verdaderas bacteriemias (N=3875).

Variable	AUC-ROC (IC95%)	Sensibilidad (%)	Especificidad (%)	LHR (+)	LHR (-)	Índice de Youden
Tiempo de positividad en horas de hemocultivos para predecir contaminantes	0,74 (0,72 - 0,76)	85,0	63,0	2,30	0,23	16,5
Microorganismos específicos						
Estafilococo coagulasa negativo	0,72 (0,70 - 0,74)	84,8	62,1	2,23	0,24	16,5
*Stenotrophomonas maltophilia*	0,61 (0,53 - 0,70)	50,0	70,0	1,66	0,71	24,5
*Candida* spp.	0,80 (0,75 - 0,84)	72,9	83,3	4,36	0,32	31,5

AUC: área bajo la curva; ROC: característica de funcionamiento del receptor; LHR: Likelihood ratio; IC95%: intervalo de confianza del 95%.

### Fiebre y antibióticos al momento de toma de muestra de hemocultivos

Se pudo obtener información de una subpoblación del estudio (n=440), de los cuales 410/440 pacientes recibieron antibióticos previos a la toma de hemocultivos, estos pacientes presentaron tiempos de positividad, más prolongados que aquellos que no recibieron antibióticos previos (14,2 horas, RIC: 11,1 - 20,1 y 10,8 horas, RIC: 9,4 - 14,2; p=0,004, respectivamente). Así mismo, en una subpoblación del estudio (n=441), observamos que 376/441 pacientes presentaron fiebre (temperatura >38,3ºC) al momento de la toma del hemocultivo, observando que la mediana de tiempo de positividad en los hemocultivos fue menor que en aquellos que no presentaron fiebre al momento de la toma de muestra (13,9 horas, RIC: 10,6 - 18,9 y 14,9 horas, RIC: 11,8 - 31,6; p=0,039, respectivamente).

## DISCUSIÓN

Encontramos que la mediana de tiempo de positividad en hemocultivos con verdaderas bacteriemias fue inferior de manera estadísticamente significativa que en los hemocultivos con contaminantes. Además, nuestros hallazgos muestran que un tiempo de positividad de hemocultivos mayor de 16,5 horas puede predecir la presencia de hemocultivos con contaminantes, con una sensibilidad y especificidad de 85% y 63%, respectivamente. Asimismo, el tiempo de positividad mostró capacidad discriminante para identificar candidemias (sensibilidad: 73%; especificidad: 83%) y bacteriemias por *Stenotrophomonas maltophilia* (sensibilidad: 50%; especificidad: 70%).

El tiempo de positividad de hemocultivos puede ayudar a distinguir entre una verdadera bacteriemia del aislamiento de microorganismos contaminantes. Ruiz-Giardín JM, *et al*. reportó que el tiempo de positividad de hemocultivos superior a 14,7 horas tuvo una sensibilidad de 90% y una especificidad de 63% con un AUC/ROC de 0,80 para predecir la detección de hemocultivos con contaminantes ^13^. Del mismo modo, Morioka S. *et al*. reportaron que el tiempo de positividad superior a 20 horas presentó una sensibilidad de 86% para predecir contaminación por estafilococo coagulasa negativo ^14^. Se presume que el tiempo de positividad de los hemocultivos podría ser un indicador de carga bacteriana. Algunos estudios han descrito una correlación inversa entre el tiempo de positividad y la concentración bacteriana en los hemocultivos ^15^. También se ha reportado que la administración de antibióticos previos a la toma del hemocultivo retrasa los tiempos de positividad ^13^^,^^16^^-^^18^, probablemente secundario al efecto reductor de la concentración bacteriana. Así mismo, un tiempo de positividad muy corto en bacteriemias por *K. pneumoniae*, se asoció con mayor mortalidad ^19^, presumimos que esta asociación con mortalidad, sea debido a una gran carga bacteriana.

La mayoría de estudios han observado que entre un 93 y 98% de verdaderas bacteriemias, fueron detectadas en las primeras 48 horas de incubación ^17^^,^^20^^-^^23^, y solo el 0,1% de verdaderas bacteriemias se aislaron después de 4 días de incubación ^24^. En nuestro estudio, el 92,2%, 99,2% y 100% de verdaderas bacteriemias se aislaron en un tiempo transcurrido menor a 2, 4 y 5 días hasta la positividad, respectivamente. Estos hallazgos sugieren que, con el equipo «BACT/ALERT®», un tiempo de incubación de 4 días podría ser suficiente para el diagnóstico de bacteriemias verdaderas, lo que permitiría reducir los aislamientos de microorganismos contaminantes, los cuales se han asociado a un incremento de costos hospitalarios ^25^^,^^26^. Hallazgos similares han sido reportados con otros sistemas de incubadoras automatizadas como «BACT/ALERT®» virtuo ^24^, Difco ESP ^27^ y Accumed ESP-384 ^28^.

También se ha planteado utilizar los tiempos que transcurren hasta la positividad de hemocultivos, como una estrategia de control de antimicrobianos. Pardo *et al*. detectaron *Pseudomonas aeruginosas*, *E. Coli*, *K. pneumoniae* y *E. cloacae* en las primeras 48 horas de incubación ^17^, y proponen que, si un hemocultivo es negativo a las 48 horas, las probabilidades de que sea una verdadera bacteriemia es mínima, debido a su alto valor predictivo negativo, muy similar al hemocultivo negativo a los 5 días; esto podría ser una oportunidad para reducir el uso innecesario de antibióticos principalmente cuando el paciente se encuentra estable y no haya recibido antibióticos previos a la toma del hemocultivo. Hallazgos similares fueron reportados por otros autores ^13^^,^^24^.

En nuestro estudio observamos diferencias en los tiempos de positividad de hemocultivos según el microorganismo aislado, principalmente en dos patógenos relevantes: *Candida* y *Stenotrophomonas maltophilia*, los cuales presentaron tiempos de positividad de 38,61 y 23,90 horas respectivamente, de modo tal que, si los hemocultivos fueron positivos más allá de las 24 horas de incubación, la posibilidad de aislar *Stenotrophomonas maltophilia* fue de un 50%; en cambio, si los hemocultivos fueron positivos más allá de las 39 horas, la posibilidad de aislar *Candida* fue de un 50%. Esto podría presentar particular relevancia en la decisión terapéutica, ya que debido a los múltiples mecanismos de resistencia intrínseca a los antibióticos que posee *Stenotrophomonas*, existe una alta posibilidad de iniciar antibioticoterapia inapropiada, la cual se asocia a una mayor mortalidad (61% vs 30%) y peores resultados ^29^^,^^30^.

Asimismo, es posible que la etiología de las bacteriemias pueda ser distinta según grupos etarios, y esto influya también en los tiempos de positividad de los hemocultivos. A diferencia de nuestro estudio que incluyó a pacientes ≥18 años, los tiempos de positividad de hemocultivos tuvieron una mediana de 11,2 horas y 12,6 horas en pacientes pediátricos de 0 a 1 años y de 1 a 15 años, respectivamente ^21^. Este tiempo de positividad, es inferior a lo observado en nuestro estudio, donde la mayoría de pacientes fueron adultos.

Se han evaluado algunos marcadores inflamatorios para predecir verdaderas bacteriemias en hemocultivos positivos. De ellos, la procalcitonina presentó el mejor desempeño con un AUC ROC de 0,79, sensibilidad de 76% y especificidad de 69% con un punto de corte de 0,5 ng/ml ^31^. Seguido por la proteína C reactiva, con un AUC ROC de la PCR de 0,64 y sensibilidad de 87% a un punto de corte de 9 mg/l; y la leucocitosis >12 000/mm^3^ con un AUC ROC de 0,69, y una sensibilidad de 65,5% ^32^. Por lo tanto, en comparación con estos marcadores inflamatorios, el tiempo de positividad de los hemocultivos observado en nuestro estudio (AUC ROC de 0,74, sensibilidad de 85% y especificidad de 63%) podría ser un mejor predictor de verdaderas bacteriemias que la proteína C reactiva y la leucocitosis, y semejante a la procalcitonina.

Este estudio tiene ciertas limitaciones. La principal fue la naturaleza retrospectiva del estudio, ya que no permitió determinar otras variables como severidad del cuadro, foco infeccioso, antibióticos recibidos y desenlace, entre otros. Tampoco fue posible cuantificar por peso el volumen de sangre inoculado en los frascos de hemocultivo y cuanto fue inoculado, el tiempo que pasó entre la extracción de la sangre y la inoculación, además del tipo de antibiótico, dosis y tiempo de tratamiento, lo cual puede afectar su desempeño. Otra limitación fue que aproximadamente 3434 muestras no presentaron información con respecto a la variable “fiebre al momento de toma de hemocultivo” y es posible que un gran porcentaje de estos hayan presentado fiebre o no, lo cual podría afectar los resultados. De la misma forma, no hay información para la mayoría de la población con respecto a la toma de antibióticos antes del hemocultivo lo cual podría afectar en gran medida los resultados. Finalmente, todos los hemocultivos fueron analizados con el equipo BacT Alert y estos hallazgos no deben ser extrapolados a otros equipos automatizados.

Este estudio demostró que los tiempos de positividad de hemocultivos pueden predecir verdaderas bacteriemias de contaminantes cuando el tiempo de positividad es superior a 16,5 horas. Asimismo, el tiempo de positividad demostró ser útil para predecir bacteriemias por *Candida* y *Stenotrophomonas maltophilia*. Conocer la utilidad diagnóstica de este método ayudaría al personal de salud a tomar mejores decisiones respecto al tratamiento del paciente y evitar costos hospitalarios innecesarios.
